# Surgical extravascular implantable cardioverter-defibrillator lead implant when transvenous therapy is undesirable

**DOI:** 10.1016/j.hrcr.2026.06.003

**Published:** 2026-06-10

**Authors:** Andor Bik, Hans Römers, Jippe Balt, Edgar Daeter, Martin Swaans, Lucas Boersma

**Affiliations:** 1 Department of Cardiology, St. Antonius Hospital, Nieuwegein, The Netherlands; 2 Department of Cardiology, Amsterdam University Medical Centers, University of Amsterdam, Amsterdam, The Netherlands

**Keywords:** Extravascular defibrillator, Cardiac resynchronization therapy, Device-related complications, Implantable defibrillator, Dual device implant

**Key Teaching Points**

• The concomitant use of an extravascular implantable cardioverter-defibrillator and a biventricular endocardial–epicardial pacing system seems to be technically feasible.
• This strategy is highly individualized and may not be suitable for the broader patient population.
• Extensive intraprocedural and longitudinal testing is necessary during implantation and follow-up.

## Introduction

Implantable cardiac defibrillators have become a cornerstone in the prevention of sudden cardiac death. Nowadays, there are transvenous and epicardial lead-based systems and subcutaneous defibrillator technologies, including the subcutaneous implantable cardioverter-defibrillator (ICD)^1^ and the emerging extravascular ICD (EV-ICD). Transvenous systems are associated with several lead-related complications, including lead fracture, infection, tricuspid valve regurgitation, and venous thrombosis. These complications may significantly affect patient outcomes and quality of life. ICD systems without leads in the vasculature are associated with a lower risk of these complications and may offer good alternatives when transvenous therapy becomes problematic. We describe 2 patients requiring cardiac resynchronization therapy (CRT) and secondary prevention of ventricular arrhythmias, in whom implantation of a transvenous shock lead was considered undesirable, and in whom a surgically positioned EV-ICD lead was used as an alternative strategy.

According to manufacturer specifications, the EV-ICD is not intended to be used in combination with bradycardia pacing therapy, mainly because of the potential risk of atrial signal oversensing leading to inappropriate shocks. However, emerging evidence suggests that selected combinations may be feasible. Previous case reports have described the use of an EV-ICD in combination with a dual-chamber transvenous or leadless pacemaker, as well as surgical implantation of an EV-ICD shock lead during open-heart surgery.^2^, ^3^, ^4^, ^5^

## Case presentation

### Case 1

A 64-year-old man with a history of genetically inherited hypertrophic obstructive cardiomyopathy presented with progressive symptoms of right-sided heart failure. He suffered an out-of-hospital cardiac arrest owing to ventricular fibrillation in 2008 followed by ICD implantation, with recurrent ventricular tachyarrhythmias and appropriate device therapy. In 2021, he underwent an upgrade to a CRT-defibrillator (CRT-D) because of cardiomyopathy and pacing indications. His medical history included persistent atrial fibrillation with multiple electrical cardioversions, leading to suboptimal CRT response for which His-bundle ablation was performed in January 2025, rendering the patient fully pacing dependent.

Several months later, he developed severe symptomatic tricuspid regurgitation. Echocardiography demonstrated interference of the right ventricular (RV) lead with the anterior tricuspid leaflet, whereas annular dilatation was only mild.

**Figure 1:**
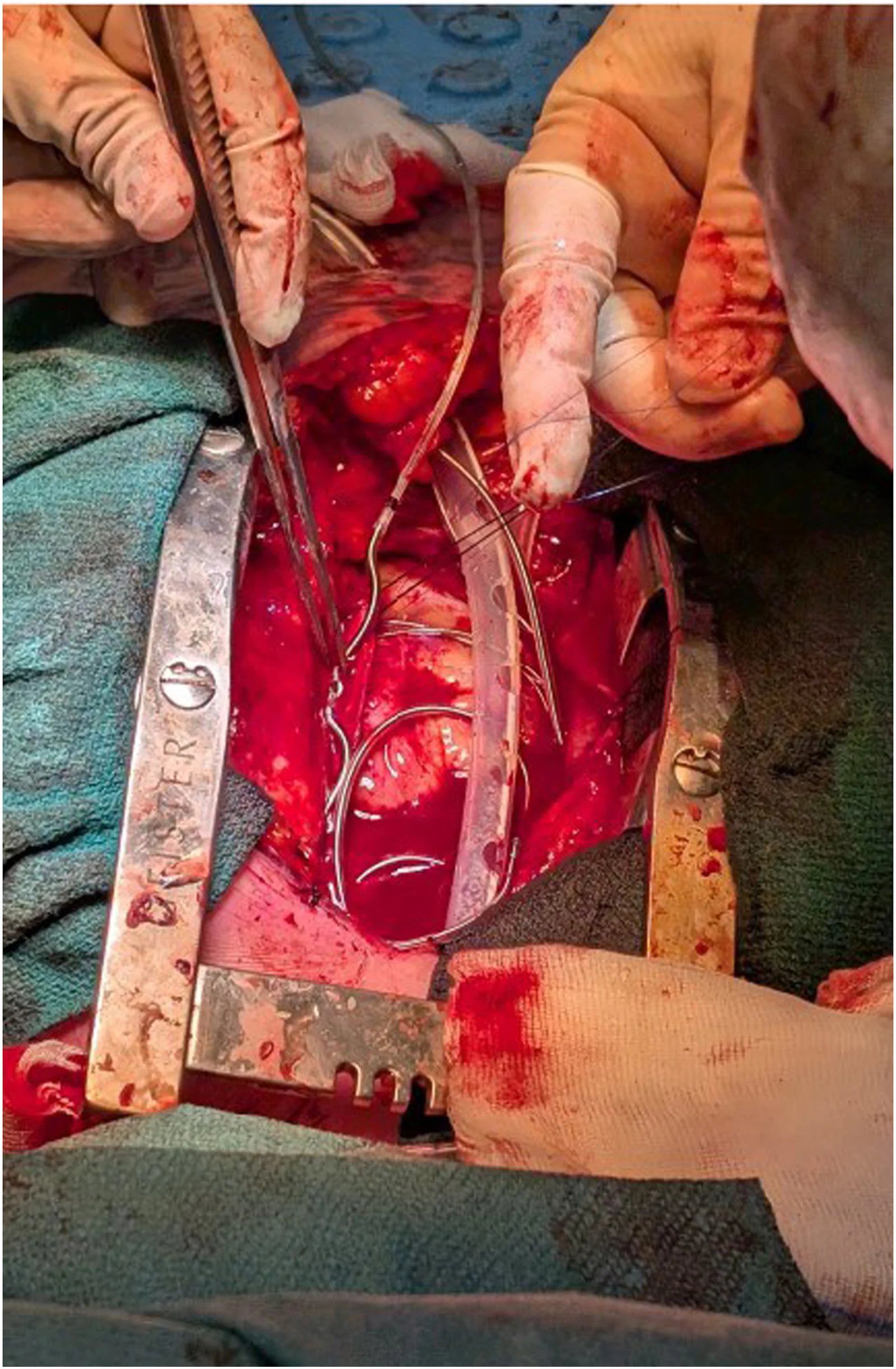
Open-chest placement of the extravascular lead directly on the pericardium.

**Figure 2:**
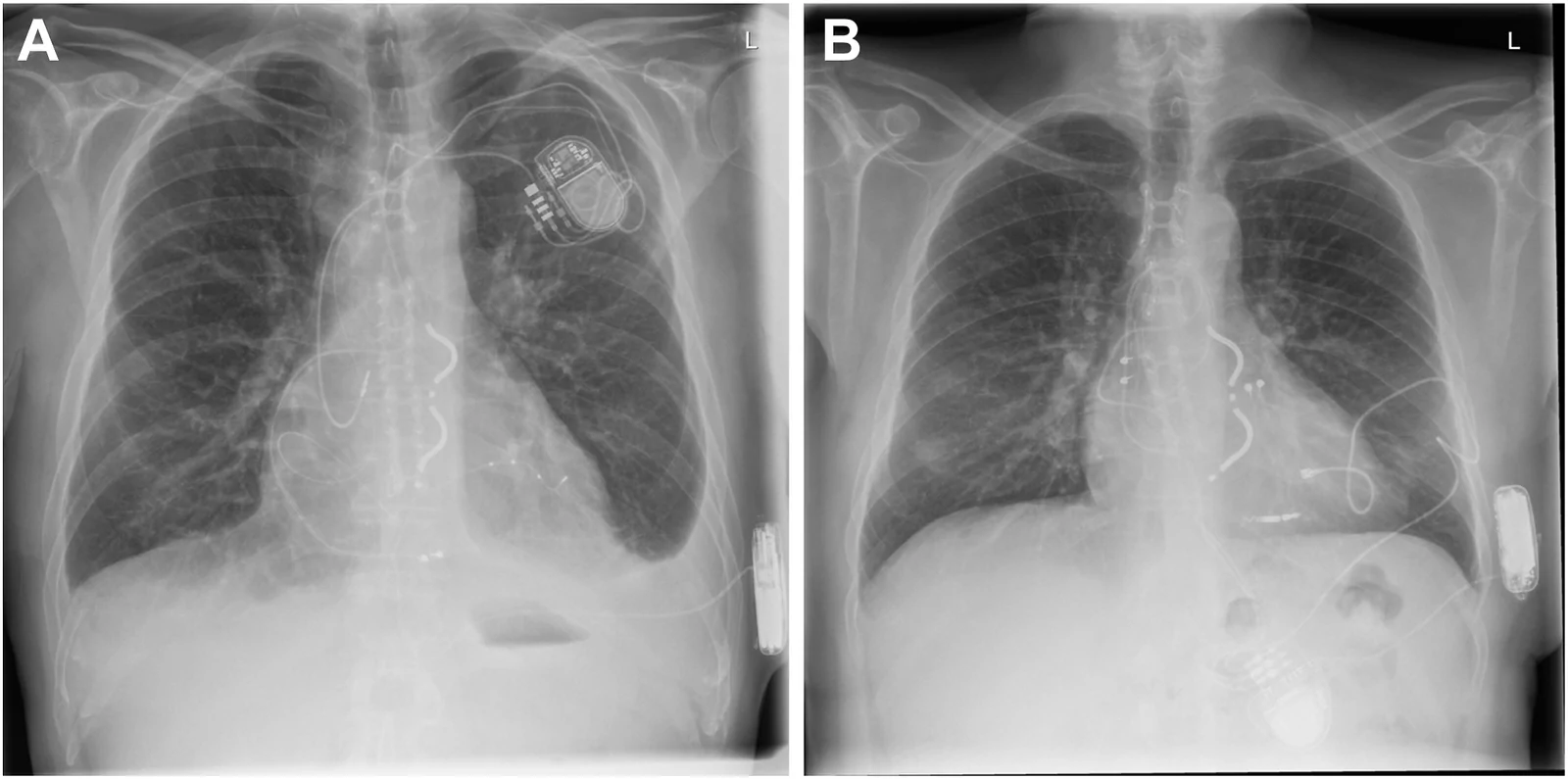
**A and B:** Radiograph after implant for both patients.

The transvenous shock lead was extracted using laser techniques. A new RV lead was implanted under transesophageal echocardiography to avoid tricuspid leaflet interference. However, this did not result in clinical improvement of the severe tricuspid regurgitation. Therefore, tricuspid valve repair with an annular ring and neo-chordae implantation was performed through midsternal thoracotomy with removal of the transvenous RV shock lead. Given that cardiac resynchronization remained indicated, a mandatory RV lead to trigger pacing was obtained with a sutured epicardial electrode in addition to the existing left ventricular (LV) lead and connected to a Biotronik Edora HF-T QP device. To enable secondary prevention tachyarrhythmia therapy with both antitachycardia pacing and shock option, an EV-ICD shock lead was sutured to the pericardium during the same procedure (Figure 1). Intraoperative measurements showed excellent sensing (R-wave amplitude 6.8 mV) without P-wave sensing. Of note, although the lead was left on the pericardium with the ring electrodes to the left and the coils to the right side of the patient, the radiograph after surgery showed the lead had flipped 180 degrees (Figure 2A). 5 days later, during a second surgical procedure, the shock lead was tunneled to a left lateral pocket and connected to a Medtronic Aurora™ EV-ICD generator. Adequate sensing was confirmed (R-wave amplitude 2.7 mV), with only marginal atrial signal detection. Defibrillation testing was successful at 30 J with a shock impedance of 41 Ω. At follow-up, R-wave amplitude improved to 3.6 mV, and ventricular capture was obtained at 4.0 V/4.0 ms. 1 episode of nonsustained ventricular tachycardia was recorded. Device interrogation showed 2% atrial pacing and 94% biventricular bipolar pacing without oversensing by the EV-ICD. At the second follow-up, 5 months after implantation, the patient is recovering well, with improving exercise capacity. EV-ICD interrogation revealed an R-wave amplitude of 5.3 mV and no recorded events. The CRT device demonstrated 98% biventricular pacing with device parameters, including stable pacing thresholds, sensing, and lead impedance.

### Case 2

A 58-year-old man with ischemic cardiomyopathy (LV ejection fraction 25%–29%) after a previous anterior myocardial infarction was referred because of recurrent system infection requiring system extraction. He had received a dual-chamber ICD for secondary prevention in 2009, later upgraded to CRT-D with an epicardial LV lead because of progressive systolic heart failure and left bundle branch block. His history was notable for numerous pocket revisions, seromas, hematomas, and infections over more than a decade.

An initial attempt at complete transvenous lead extraction using laser and TightRail techniques was aborted because of hemodynamic instability and significant blood loss. During a subsequent open-heart surgery, the right atrial and RV shock leads were largely removed, although a small portion of the RV shock coil could not be removed because of dense adhesions to the RV wall. Given the patient’s clear hemodynamic benefit from CRT, a fully epicardial pacing system was implanted consisting of right atrial, RV, and LV epicardial leads connected to a Medtronic Serena CRT-P device. In addition, an EV-ICD shock lead was sutured to the pericardium over the RV with the remaining lead and connector left subcutaneously at the substernal incision site. Sensing was adequate, with an R-wave amplitude of 1.6 mV. Again, although the lead was left on the pericardium with the ring electrodes to the left and the coils to the right side of the patient, the radiograph after surgery showed the lead had flipped 180 degrees (Figure 2B). During a subsequent procedure several days later, the shock lead was tunneled from the abdominal incision to a left lateral pocket and connected to an Aurora EV-ICD generator. R-wave amplitude remained adequate without P-wave sensing, and defibrillation testing was successful at 30 J with ventricular capture obtained at 4.0 V/4 ms. At follow-up of the EV-ICD, all electrical measurements remained stable whereas no atrial oversensing was observed, even at maximal pacing output. Interrogation of the CRT-P device showed atrial pacing of <0.1% and ventricular pacing 98.4%, with 100% biventricular pacing. 5 months after implantation, at the second follow-up visit, patient 2 exhibits good recovery with slowly improving physical exercise tolerance. Evaluation of the EV-ICD revealed an R-wave amplitude of 3.4 mV and no detected events. The CRT system demonstrated 90.2% biventricular pacing with stable pacing thresholds, sensing, and lead impedance.

## Discussion

In patients with an indication for a CRT-D device with a contraindication for an endovascular shock lead, combining an EV-ICD with an epicardial CRT-pacing system may be a valuable alternative in carefully selected patients. Currently, manufacturer recommendations advise against concomitant EV-ICD and pacemaker therapy. Despite this, existing case reports indicate that this combination may represent a safe and effective option in carefully selected patients. During a standard implantation, the EV-ICD lead is tunneled percutaneously to its substernal position. Obtaining a sufficiently large R wave while avoiding P-wave detection can be challenging but is essential to prevent inappropriate shocks while securing effective arrhythmia detection and treatment. The open-chest surgery allows for direct suturing of the lead onto the ventricular pericardium over the RV wall, thereby minimizing the risk of atrial oversensing. Of note, in both patients, despite proper positioning and fixation, the EV-ICD shock lead had rotated during thoracic closure, resulting in a reversed left-sided orientation of the coils.

However, during the subsequent EV-ICD implantation, several days after the cardiac surgery, electrical performance had remained satisfactory and defibrillation testing was successful at 30 J allowing a safety margin of 10 J. We opted for implantation of the EV-ICD device as a staged second procedure to enable stabilization of the heart after surgery and additional evaluation of sensing performance and defibrillation threshold to ensure continued stability and secure lead placement. The reversal of the coil position to the left of the heart emphasizes the importance of adequate surgical fixation and avoiding rotational force on the lead while closing the chest and positioning the lead under the skin below the xiphoid.

**Figure 3:**
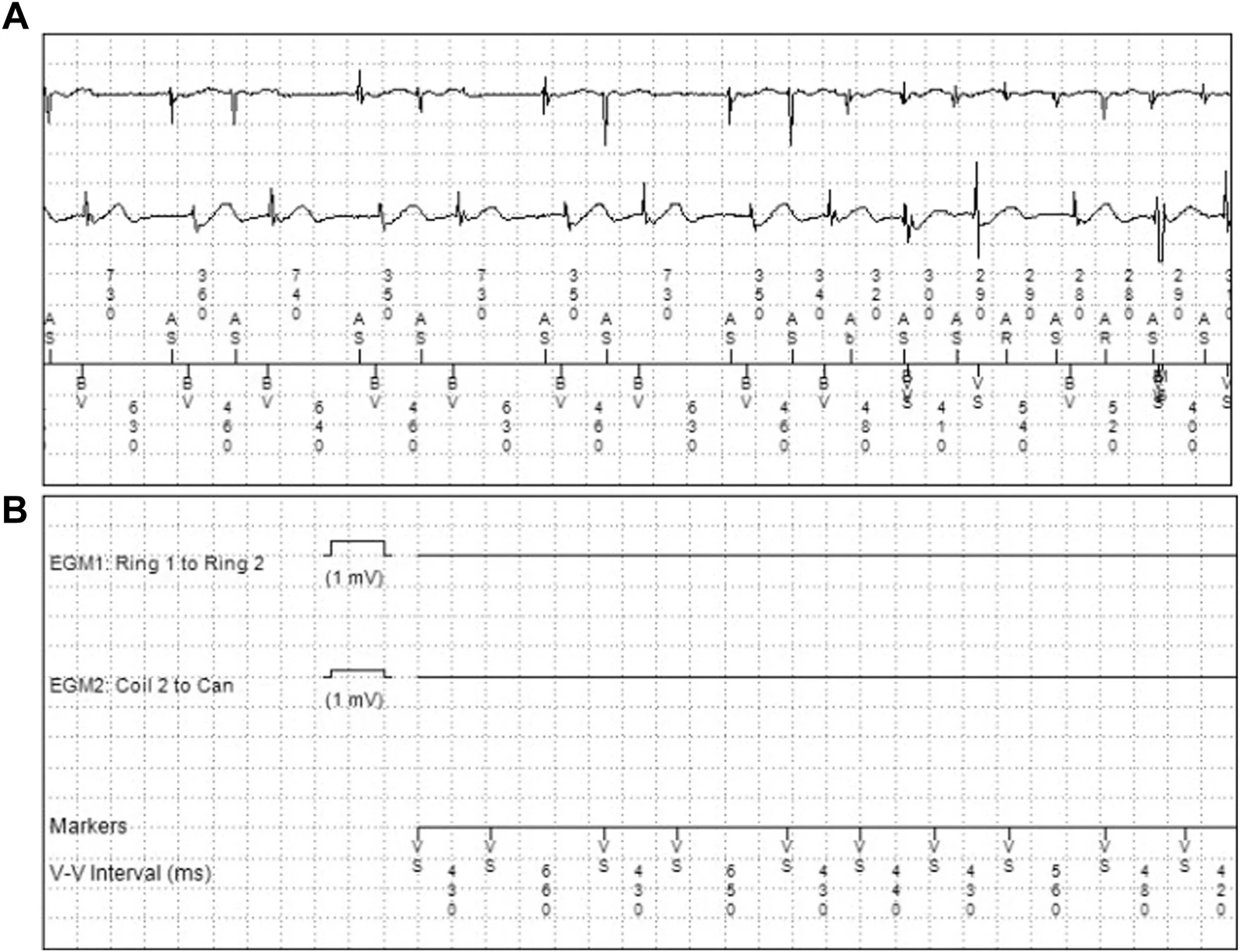
**A:** An electrogram of a short burst of atrial tachycardia in a cardiac resynchronization therapy device. **B:** A corresponding electrogram of sensing ventricular rhythm in the marker channel in an extravascular implantable cardioverter-defibrillator without atrial oversensing.

During and after implantation, comprehensive testing of all pacing configurations is mandatory, particularly bipolar and unipolar lead settings of the CRT device at maximal output, to assess the risk of oversensing (Figure 3A and 3B). This evaluation is essential to ensure patient safety in the event of an automatic safety switch to unipolar pacing involving 1 or more pacing leads. Potential consequences include the need to disable automatic switching from bipolar to unipolar pacing. The EV-ICD was programmed with postshock pacing deactivated, given that pacing therapy was delivered by the concomitant CRT device. Follow-up of both patients’ devices revealed no abnormalities. Measurements remained stable, and no events occurred that necessitated device reprogramming or clinical intervention.

Both patients are connected to remote monitoring for both devices for extensive monitoring performance and early detection of inappropriate device function.

## Conclusion

In patients in need of sudden cardiac death prevention and CRT with a contraindication to transvenous lead use, surgical positioning of an EV-ICD lead and epicardial pacing leads provides a viable alternative. Adequate positioning and fixation of the EV-ICD lead are essential to achieve optimal R-wave sensing while minimizing atrial oversensing. Device performance should be tested under extreme pacing conditions to avoid oversensing and inappropriate shock. Although electrical performance during short-term follow-up was excellent, further monitoring is required to determine long-term stability and efficacy of surgically implanted EV-ICD shock leads.

## Funding Sources

This research did not receive any specific grant from funding agencies in the public, commercial, or not-for-profit sectors.

## Disclosures

J. Balt is a consultant for Abbot. L. Boersma is a consultant for Medtronic, Boston Scientific, Philips, Zoll, Biosense Webster, and Abbott. The other authors have no conflicts of interest to disclose.
